# Analysis of the current situation of female induced abortion under China’s “three-child policy”

**DOI:** 10.1371/journal.pone.0351411

**Published:** 2026-07-01

**Authors:** Lingna Huang, Jinwen Zhang, Kuncan Chen, Kairui Lin, Xin Wu, Yushan Hong, Yihua Ni, Xiujuan Chen

**Affiliations:** 1 Department of Gynecology and obstetrics, Fujian Maternity and Child Health Hospital, College of Clinical Medicine for Obstetrics & Gynecology and Pediatrics, Fujian Medical University, Fuzhou, Fujian, China; 2 School of Basic Medicine Sciences, Fujian Medical University, Fuzhou, Fujian, China; Shanghai Institute for Biomedical and Pharmaceutical Technologies, CHINA

## Abstract

**Objective:**

To assess the changes in the characteristics of women undergoing induced abortion and their contraceptive choices before and after the implementation of the “three-child” policy, and to inform strategies for improving reproductive health services in China.

**Methods:**

We conducted a retrospective comparative study at a tertiary maternity hospital in Fujian, China. Women who underwent induced abortion between 31 May 2019 and 30 May 2020 (pre-policy period) and those who underwent the procedure between 31 May 2023 and 30 May 2024 (post-policy period) were included. We used R software (version 4.3.2) to compare contraceptive use and method choices prior to the abortion, as well as the primary reasons for unintended pregnancy between pre- and post-policy periods.

**Results:**

In the pre-policy period, non-use of contraception was the leading cause of unintended pregnancy (43.38%), primarily attributed to a perception of low pregnancy risk. Among contraceptive users, condoms were the most common method (26.47%). Post-policy, the rate of contraceptive non-use decreased to 32.19%. However, reliance on low-efficacy methods persisted, with condoms (43.49%) and withdrawal (23.51%) remaining predominant.

**Conclusion:**

Non-use of contraception and reliance on low-efficacy methods remained the primary contributors to unintended pregnancies among women undergoing induced abortion both before and after the three-child policy. Higher educational attainment was associated with lower contraceptive failure risk. These findings underscore the urgent need to improve access to effective contraceptive methods and strengthen reproductive health education to reduce unintended pregnancies and safeguard women’s reproductive health.

## Introduction

Fertility rates are negatively correlated with socioeconomic development globally, even in regions experiencing rapid economic growth [1,2]. Low fertility has become a major demographic concern in many countries, including China, a populous nation similarly affected by this trend. According to data from the National Bureau of Statistics, China’s fertility rate has been steadily declining since the early 1990s [3]. This persistent decline has accelerated population aging and contributed to a shrinking labor force [4]. In response to these adverse trends, the Chinese government has introduced measures to encourage marriage and childbirth, most notably the “two-child” policy in 2015 and the “three-child” policy in 2021. Nevertheless, these policies still face substantial challenges in effectively stimulating population growth. Notably, regional data reveal an increase in unintended pregnancies and induced abortions [5,6]; younger and unmarried women, and those with a history of multiple abortions are overrepresented in these populations.

The high rate of induced abortions contributes a major threat to women’s reproductive health. It is close associated with unintended pregnancy and inadequate use of contraceptive methods, and can lead to complications such as intrauterine adhesions, chronic pelvic inflammatory disease, abdominal pain, psychological distress, secondary infertility, and even death [7–9]. This situation reflects not only deficiencies in contraceptive counselling and services but also shifts in reproductive decision-making. Elucidating the determinants of the high abortion rate is essential to understanding the current landscape of induced abortion among Chinese women in the context of the new policy.

This study aims to compare contraceptive preferences and patterns of induced abortion between the pre- and post-“three-child” policy cohorts, and to investigate the root causes of unintended pregnancies after the policy’s implementation. By generating evidence to support health authorities in developing contraceptive service strategies, this study is expected to enhance the theoretical framework linking fertility policies to individual reproductive behaviors. Ultimately, it will contribute to reducing the incidence of non-medically indicated induced abortions.

## Methods

### Study design and participants

This study included patients undergoing induced abortion for unintended pregnancy at Fujian Maternity and Child Health Hospital. For the post-policy cohort, all consecutive patients who underwent induced abortion between May 31, 2023, and May 30, 2024 were screened; those who fulfilled the eligibility criteria were prospectively enrolled, and their data were entered into a clinical database. For comparison, the pre-policy cohort was formed by retrospectively reviewing the records of all consecutive patients who underwent induced abortion between May 31, 2019, and May 30, 2020. To minimize selection bias, no sampling was performed; the study aimed to capture the entire population of eligible patients within the defined time periods. Patients with missing key variables or who did not meet the inclusion criteria were excluded based on predefined criteria.

### Eligibility criteria

Inclusion criteria were: (1) first-trimester pregnancy (gestational age < 10 weeks); (2) viable intrauterine pregnancy confirmed by ultrasound; and (3) voluntary termination of pregnancy, with no contraindication to induced abortion, and provision of written informed consent for the surgical procedure. Exclusion criteria were: (1) spontaneous abortion or abnormal pregnancy, including hydatidiform mole and missed miscarriage; (2) induced abortion due to maternal medical reasons (e.g., congenital heart disease, hematological diseases, acute infection, malignant tumors, chronic inflammatory diseases), fetal genetic abnormalities, or other serious personal health conditions.

### Data collection

A standardized transvaginal ultrasound examination was performed on every woman prior to the procedure. For the post-policy cohort, individualized counseling sessions were conducted in a private setting with no unrelated individuals present. Medical staff explained the study objectives, significance, content of the questionnaire, and instructions for completion. After providing consent, participants completed an anonymous self-administered questionnaire on-site. For pre-policy cohort, trained medical personnel retrospectively abstracted relevant data from the medical records to ensure accuracy and confidentiality. The information collected included demographic characteristics (age, marital status, place of origin, occupation, and educational level) and reproductive history (number of prior induced abortions, contraceptive methods used, reasons for contraceptive failure, and future childbearing intentions).

### Statistical analysis

All statistical analyses were performed using R software (http://www.R-project.org; Version 4.2.1). Categorical variables are presented as frequencies (percentages), continuous variables are expressed as mean ± standard deviation. Between-group differences in continuous variables were assessed using the independent-samples t-test for normally distributed data and Mann-Whitney U test for non-normally distributed data, as appropriate. Categorical variables were compared with the chi-square test. To explore factors associated with contraceptive failure, a multivariable binary logistic regression model was fitted, with age, educational level, marital status, and household registration included as independent variables. A two-sided p < 0.05 was considered statistically significant. For multiple comparisons, p-values were adjusted using the Benjamini–Hochberg procedure to control the false discovery rate, with an adjusted p < 0.05 regarded as significant.

### Ethical considerations

This study was conducted in accordance with the Helsinki Declaration (as revised in 2013). The protocol was approved by the Medical Ethics Committee of Fujian Maternal and Child Health Hospital (approval number: 2023KY262), and all procedures complied with institutional and national regulations. All participants in the “post-policy” cohort provided written informed consent. For pre-policy cohort, the ethics committee approved the use of verbal informed consent obtained by telephone to minimize participant burden.

## Results

### Demographic characteristics

A total of 1,000 cases were initially identified for the “pre-policy” cohort. After excluding cases with missing data and applying predefined exclusion criteria, 869 eligible women remained. For the “post-policy” cohort, 1,400 women were initially screened, 1,221 were included in the final analysis. A comprehensive summary of baseline characteristics are presented in Table 1.

**Table 1:** Demographic characteristics of participants in pre- and post-policy cohort.

	Three child policy			
Variables	Before the policy N = 869	After the policy N = 1221	statistics	p-value
**Age**	30.49 ± 5.55	31.32 ± 5.78	3.296	0.001
< 18	1(0.11%)	5(0.41%)		
18-35	697(80.21%)	922(75.51%)		
> 35	171(19.68%)	294(24.08%)		
**Marital status**				0.412
Widowed	2 (0.23%)	0 (0.00%)		
married	682 (78.48%)	961 (78.71%)		
unmarried	182 (20.94%)	253 (20.72%)		
divorce	3 (0.35%)	7 (0.57%)		
**Resident place**			97.78	<0.001
Rural areas in other counties, cities, and districts	10 (1.15%)	28 (2.29%)		
Cities in other counties, cities, and districts	82 (9.44%)	12 (0.98%)		
Rural areas in this city	29 (3.34%)	17 (1.39%)		
City within this district	748 (86.08%)	1,164 (95.33%)		
**Education level**			2.65	0.266
Primary school or below	140 (16.11%)	169 (13.84%)		
Middle school	420 (48.33%)	587 (48.08%)		
College degree or above	309 (35.56%)	465 (38.08%)		
**Whether having chronic disease**			7.14	0.008
No	834 (95.97%)	1,197 (98.03%)		
Yes	35 (4.03%)	24 (1.97%)		

The mean age was 30.49 ± 5.55 years in the pre-policy cohort and 31.32 ± 5.78 years in the post-policy cohort. Women who underwent induced abortion after the implementation of the three-child policy were significantly older than their pre-policy counterparts; and 294 (24.08%) of post-policy women were over 35 years old. The majority of women in both cohorts were married; Urban household registration (hukou) predominated in both groups, and the proportion increased significantly after the policy change, reaching 95.33% (1,164/1,221) in the post-policy cohort. Nearly half (48.33%) of participants in both cohort (48.08%in pre-policy cohort vs. 48.08% in post-policy cohort) had completed middle school, whereas a colleague degree or higher was held by 35.56% and 38.08%, respectively. While the majority of participants in both groups were in good health, a small proportion reported pre-existing chronic diseases— 4.03% in the pre-policy cohort and 1.97% in the post-policy cohort.

### Differences of pregnancy history, contraceptive practices and reproductive intentions in the two cohorts

There were no statistically significant differences in the number of pregnancies or induced abortions between the two groups; the highest number of documented induced abortions was eight before the policy was implemented and eleven after. The most common method of terminating unwanted pregnancies was surgical abortion, which accounted for 685 cases (78.83%) in the pre-policy group and 1,012 cases (82.88%) in the post-policy group. Before the policy was implemented, 377 (43.38%) of women did not use any form of contraception; this number decreased only slightly to 393 (32.19%) after the policy was implemented. Among women undergoing induced abortion, more than 70% reported that the unintended pregnancy occurred in the absence of any contraceptive use. The majority of those using contraception relied primarily on withdrawal (114 [13.12%] before vs. 287 [23.51%] after) and condoms (230 [26.47%] before vs. 531 [43.49%] after), both of which have comparatively low efficacy [10]. As a result, the rate of unintended pregnancies caused by inappropriate contraceptive use was 224 (25.78%) before the policy implementation and 321 (26.29%) after it. Prior to the three-child policy, 122 women (14.04%) planned to have children after more than two years, while 633 women (72.84%) reported having no plans to have children at all. Following the implementation of the policy, 441 women (36.12%) planned to have children after more than two years, whereas the number of women who had no plans to have children decreased to 569 (46.60%) (Table 2).

**Table 2:** Differences of fertility history, contraceptive use, and reasons of abortion between the Two Groups.

	Three child policy			
Variables	Before the policy N = 869	After the policy N = 1221	p-value	
**Gravidity (Mean, SD)**	3.040 ± 1.473	2.950 ± 1.589	0.201	
**Parity (Mean, SD)**	1.170 ± 0.836	1.110 ± 0.880	0.103	
**Number of previous induced abortions (Mean, SD)**	1.860 ± 1.036	1.840 ± 1.100	0.648	
**Contraceptive use before abortion (n,%)**			<0.001	
Intrauterine devices (IUDS)	3 (0.35)	2 (0.16)		
Oral contraceptive pills	33 (3.80)	3 (0.25)		
Periodic abstinence	112 (12.89)	5 (0.41)		
Withdrawal	114 (13.12)	287 (23.51)		
Condoms	230 (26.47)	531 (43.49)		
Non-method users	377 (43.38)	393 (32.19)		
**Reasons of pregnancy (n,%)**			0.878	
Method failed	7 (0.81)	12 (0.98)		
Improper use	224 (25.78)	321 (26.29)		
Not using contraceptive methods	638 (73.42)	888 (72.73)		
**Method of abortion (n,%)**			0.022	
Surgical abortion	685 (78.83)	1,012 (82.88)		
Medical abortion	184 (21.17)	209 (17.12)		
**Fertility intention (n,%)**			<0.001	
< 7 months	14 (1.61)	21 (1.72)		
7–12 months	41 (4.72)	21 (1.72)		
13–24 months	15 (1.73)	152 (12.45)		
> 24 months	122 (14.04)	441 (36.12)		
No plans at the moment	633 (72.84)	569 (46.60)		
Not sure	44 (5.06)	17 (1.39)		

No statistically significant differences were observed between the two cohorts in gravidity or the number of prior induced abortions. Surgical abortion was the predominant method of pregnancy termination in both groups (78.83% pre-policy vs. 82.88% post-policy). The proportion of women who did not use any contraceptive method decreased from 43.38% (377/869) to 32.19% (393/1,221) after the policy, yet unintended pregnancies in the absence of contraception remained common. Among contraceptive users, the most frequently reported methods were withdrawal (coitus interruptus) and condoms, both of which are associated with relatively low real-world effectiveness. Their use increased significantly after the policy: withdrawal from 13.12% to 23.51%, and condoms from 26.47% to 43.49%. The percentage of unintended pregnancies attributable to contraceptive failure or incorrect/inconsistent use remained similar (25.78% vs. 26.29%). With respect to reproductive intentions, a marked shift was observed: the proportion of women planning to conceive after more than two years rose from 14.04% to 36.12%, whereas those reporting no childbearing intention declined from 72.84% to 46.60% (Table 2).

### Factors Associated with Contraceptive Failure: Logistic Regression Analysis

Multivariate binary logistic regression analysis was performed to explore contraceptive failure with women’s age, marital status, resident place and educational attainment included in the regression model. As presented in Table 3, educational attainment emerged as a significant independent influencing factor. Compared with women educated at primary school level or below, respondents with junior and senior high school education (OR = 0.656, 95% CI: 0.506–0.849, p = 0.001) and those holding college degree or higher (OR = 0.611, 95% CI: 0.427–0.875, p = 0.007) presented markedly lower risks of contraceptive failure. No statistically significant correlations were observed between contraceptive failure and age, marital status or household registration residence (all p > 0.05).

**Table 3:** Factors associated with contraceptive failure: results of logistic regression analysis.

Variable Category	Group	Odds Ratio (OR)	95% Confidence Interval (CI) for OR
**Age**	＜**181**	–	–
	＞**35**	0.623	0.173-2.237
	**18-35**	0.578	0.167-2.007
**Education level**	Primary school or below**1**		
	College degree or above	0.611	0.427-0.875
	Middle school	0.656	0.506-0.849
**Marital Status:**	Married**1**	–	–
	Unmarried	1.167	0.904-1.505
**Resident place**	City within this district**1**	–	–
	Rural areas in this city	0.948	0.348-2.587
	Cities in other counties, cities, and districts	1.220	0.590-2.525
	Rural areas in other counties, cities, and districts	1.294	0.610-2.746
**Intercept**		–	–

^1^：Reference Group

Dependent Variable: Contraceptive Failure Status

### Reasons for non-contraceptive use and induced abortion following the three-child policy implementation

**Fig 1:**
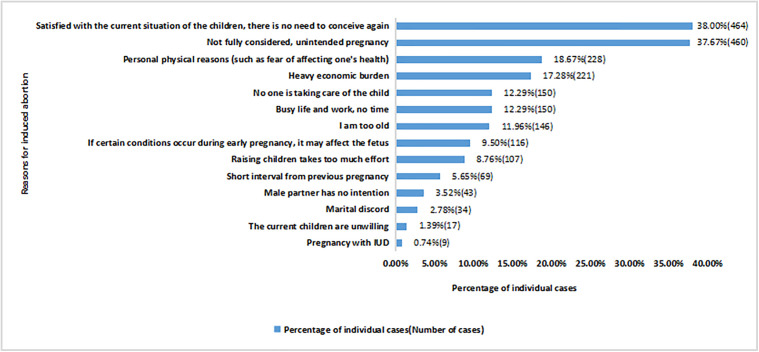
Reasons for induced abortion.

After the rollout of the three-child policy, diverse practical factors led women to terminate unintended pregnancies. The top reasons for induced abortion were satisfaction with existing family size with no willingness to have more children (464 cases, 38.0%) and failure to anticipate accidental pregnancy (460 cases, 37.67%). Heavy economic pressure (221 cases, 17.28%) and individual health risks (228 cases, 18.67%) also served as critical driving causes (Fig 1).

**Fig 2:**
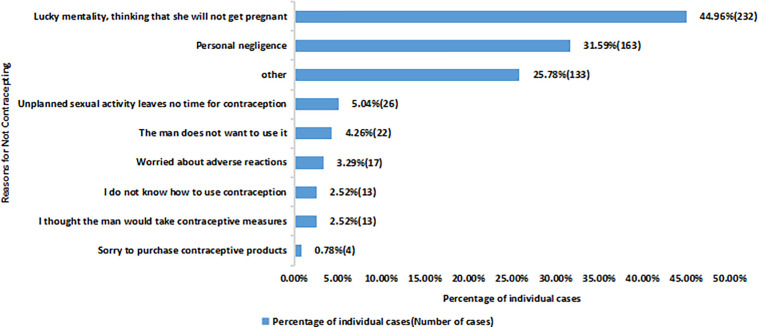
Reasons for not using contraception.

For non-adoption of contraceptive methods, subjective cognition dominated the main causes. The most common reason was the subjective judgment of low pregnancy possibility (232 cases, 44.96%), followed by personal negligence (163 cases, 31.59%) (Fig 2).

## Discussion

Our findings indicate that the majority of Chinese women seeking induced abortion are aged 18–35. Notably, 49.7% of our participants were unmarried, reflecting the growing proportion of young, childless, unmarried women undergoing the procedure. The annual number of induced abortions in China has risen to an average of 9.5 million since 2017 (China Health Statistics Yearbook, 2020 [11]). According to Smith et al. [12], the family planning program has profoundly shaped fertility decisions, contributing to the emergence and exacerbation of low fertility. Stover and colleagues [13] emphasized that fertility intentions and outcomes are influenced by family planning, contraceptive use, abortion, and infertility. Our analysis further revealed that while the abortion rate among married women of reproductive age is declining, rates among childless and unmarried women are rising—a trend consistent with other research [14]. Strengthening health education for unmarried individuals is essential to prevent secondary infertility caused by complications of induced abortion. The 2022 China Family Planning Association [15] underscores the need to extend reproductive health services to this population. Community-based contraceptive support programs, such as free contraceptives and dedicated counseling services, could more effectively help unmarried women access the protection they need.

With regard to education, the majority of participants in both groups had completed middle school. After the implementation of the three-child policy, the proportion of patients with lower educational attainment decreased significantly, whereas the share with a college degree or above increased. Although this study found no significant educational differences between groups, higher education is generally associated with lower fertility aspirations. Kreyenfeld et al. [16,17] found across 12 European countries that fertility aspirations were higher among less-educated women and declined with increasing education. Similarly, Chen’s study in Taiwan [18] identified rising female education as a key factor associated with declining fertility intentions. China’s expanding higher education has raised average educational attainment, a trend that correlates with lower fertility intentions [19]. While our regression analysis showed that higher education was independently associated with a lower risk of contraceptive failure, prior evidence suggests it is also linked to delayed childbearing and lower fertility intentions, which may indirectly contribute to induced abortion through mechanisms beyond contraceptive effectiveness.

Before the three-child policy, 43.38% of patients reported not using contraception; after the policy, this dropped to 32.19%. However, condoms and withdrawal remained the primary methods in both periods, meaning a large proportion of women continued to rely on no method or low-efficacy methods. A common reason for non-use was a perceived low risk of pregnancy. Higher educational attainment was associated with a lower likelihood of contraceptive failure, yet the underlying mechanisms remain speculative. To reduce abortions, public education on effective contraception must be strengthened, misconceptions dispelled, and contraceptive counseling integrated into post-abortion care. Authoritative online platforms providing expert-backed reproductive health information are also recommended [20].

Although more women expressed childbearing intentions after the policy, the observational design precludes causal inference. Unintended pregnancies were mainly attributed to financial constraints, health concerns, and satisfaction with current family size, underscoring that reproductive decisions are multi-dimensional. As reproduction becomes a personal choice, policy efforts move beyond lifting birth restrictions and aim to create a genuinely supportive social environment. Fertility intentions are shaped by regional, educational, psychological, and economic factors [21]; thus, targeted, localized support programs are essential.

This single-center study with an urban-skewed sample limits generalizability to rural areas, where contraceptive non-use is higher yet abortion rates among unmarried women are reportedly lower [22–24]. The follow-up period after the three-child policy is short, and the observational design cannot establish causality. Future multi-site, longitudinal studies with urban–rural representation are warranted.

## Conclusion

Young, unmarried, and childless women constitute the dominant group seeking induced abortion, with non-use or less effective meth remaining largely unchanged after the three-child policy. Higher education is associated with contraceptive failure, but it may also delay childbearing, indirectly shaping abortion dynamics. Unintended pregnancies are rooted in practical constraints and reproductive contentment, not merely policy context. These findings emphasize that reducing abortion rates requires a dual focus: delivering targeted contraceptive education and accessible services to unmarried and less-educated populations, while shifting policy emphasis from birth promotion to building a supportive child-rearing environment.
